# PRAF3 induces apoptosis and inhibits migration and invasion in human esophageal squamous cell carcinoma

**DOI:** 10.1186/1471-2407-12-97

**Published:** 2012-03-21

**Authors:** Guo-Zhen Shi, Yang Yuan, Guo-Jun Jiang, Zhi-Jun Ge, Jian Zhou, De-Jun Gong, Jing Tao, Yong-Fei Tan, Sheng-Dong Huang

**Affiliations:** 1 Institute of Cardiothoracic Surgery, Changhai Hospital, Second Military Medical University, Shanghai, PR China; 2 Department of Cardiothoracic Surgery, Yixing People's Hospital, Jiangsu, PR, China; 3 Department of Pathology, Yixing People's Hospital, Jiangsu, PR China; 4 Institute of Cardiothoracic Surgery, Changhai Hospital, 168, Changhai Rd, Shanghai, PR China; 5 Institute of Cardiothoracic Surgery, Yixing People's Hospital, 75 Tongzhenguan Rd, Wuxi, Jiangsu, PR China

## Abstract

**Background:**

Prenylated Rab acceptor 1 domain family member 3 (PRAF3) is involved in the regulation of many cellular processes including apoptosis, migration and invasion. This study was conducted to investigate the effect of PRAF3 on apoptosis, migration and invasion in human esophageal squamous cell carcinoma (ESCC).

**Methods:**

The expression of *PRAF3 *mRNA and protein in primary ESCC and the matched normal tissues (57cases) was determined by quantitative RT-PCR and Western blot. Immunohistochemical analysis of PRAF3 expression was carried out in paraffin-embedded sections of ESCC and correlated with clinical features. The role of PRAF3 in apoptosis, migration and invasion was studied in ESCC cell lines of Eca109 and TE-1 through the adenovirus mediated PRAF3 gene transfer. The effect of PRAF3 on apoptosis was analyzed by annexin V-FITC assay. The regulation of PRAF3 on migration was determined by transwell and wounding healing assay, while the cellular invasion was analyzed by matrigel-coated transwell assay.

**Results:**

We found that the expression of PRAF3 was significantly down-regulated in ESCC tissue compared with the matched normal tissue and was correlated with the clinical features of pathological grade, tumor stage and lymph node metastasis. Moreover, overexpression of PRAF3 induced cell apoptosis through both caspase-8 and caspase-9 dependent pathways, and inhibited cell migration and invasion by suppressing the activity of both MMP-2 and MMP-9 in human ESCC cell lines.

**Conclusions:**

Our data suggest that PRAF3 plays an important role in the regulation of tumor progression and metastasis and serves as a tumor suppressor in human ESCC. We propose that PRAF3 might be used as a potential therapeutic agent for human ESCC.

## Background

Esophageal squamous cell carcinoma (ESCC) is one of the most common malignant tumors in China, Japan, and southeast Africa [1,2]. Although novel surgical treatment can prolong the survival time of the patients,the 5-year survival rate of ESCC after surgery is low (ranging from 14%-22%) [3]. The major causes leading to the poor prognosis of the ESCC patients is tumor metastasis. Therefore, any insight into the mechanisms of ESCC cell progression and metastasis may provide important clues for the development of therapeutics [4].

Prenylated Rab acceptor 1 domain family member 3 (PRAF3, also known as ARL6IP5 and JWA) is a 21.6-kD microtubule-associated protein containing a prenylated Rab acceptor (PRA) motif [5,6]. Previous studies reported that PRAF3 is involved in the regulation of intracellular protein transport, DNA damage repair, oxidative stress and other functions, where PRAF3 has been shown to induce cell apoptosis and inhibit cell migration via different pathways [7-10]. In recent years, PRAF3 has gained increasing attention in tumor research. Unlike other members (PRAF1 and PRAF2) of the PRA family which promote tumor cell proliferation and migration and hence may function as oncogenes [11-13], PRAF3 is considered as a tumor suppressor since it could induce cell apoptosis and inhibit metastasis in tumors such as breast cancer, cervical cancer, melanoma and osteosarcoma [7,9,10]. However, the biological role of PRAF3 in ESCC has not been documented.

Here we set out to evaluate the role of PRAF3 in human ESCC by clinical investigation and cellular experiment. Clinical investigation showed that a down-regulation of PRAF3 expression was closely correlated with poorly differentiated grading, advanced tumor stage and lymph node metastasis of ESCC. With ESCC cell lines, we further demonstrate that overexpression of PRAF3 by adenovirus-mediated gene transfection could induce apoptosis and inhibit the migration and invasion. These results are consistent with the notion that PRAF3 is a suppressor in ESCC.

## Methods

### ESCC specimens

A total of fifty-seven primary ESCC patients that underwent esophagectomy were enrolled in this study. Tumor specimens and paired normal esophageal tissue specimens taken from a site distant from the cancerous lesion were obtained from the consenting patients, as approved by the Medical Ethics Committee of Yixing People's Hospital. None of the patients received radiotherapy or chemotherapy before surgery. Clinical and pathological data including age, gender, pathological grading, tumor location, tumor stage and lymph node metastasis were acquired from the medical records.

### Cell culture

Human ESCC cell lines Eca109 and TE-1 were purchased from the Shanghai Institute of Biochemistry and Cell Biology (Shanghai, China). Cells were maintained in RPMI1640 (Invitrogen) supplemented with 10% fetal bovine serum (Invitrogen), 100 U/ml penicillin and 100 μg/ml streptomycin, within a humidified atmosphere containing 5% CO_2 _at 37°C.

### Immunohistochemistry

We used primary ESCC tissues near the margin of the tumor and match normal tissues to asses PRAF3 expression. Sections (5 μm) of the specimens were incubated with goat anti-human PRAF3 antibody (Santa Cruz) overnight at 4°C, followed by incubation with horseradish peroxidase-conjugated donkey anti-goat antibody (Santa Cruz) for 1 hr at 37°C. Immunodetection was performed with the EnVision™ Kit (Dako), using diaminobenzidine as the chromogen. All slides were evaluated independently by two pathologists (ZJG. and JZ.) without prior knowledge of the clinical information of the patients. The expression of PRAF3 was considered positive if staining intensity was moderate or strong and the percentage of positively stained cancer cells were > 10%.

### Construction of recombinant adenovirus Ad.PRAF3 and cell infection

Recombinant adenovirus expressing human PRAF3 was constructed using the AdEasy system (Stategene). Human PRAF3 gene was amplified and ligated into pShuttleCMV plasmid. Ad.PRAF3 and Ad.Null were propagated in HEK293 cells and purified using BD Adeno-X™ virus purification kit (BD Biosciences). The activities of adenovirus were determined by plaque assay using BD Adeno-X™ rapid titer kit (BD Biosciences) according to the manufacturer's instruction. Cells from ESCC cell lines were infected with Ad.PRAF3 (or Ad.Null) at a MOI of 100 for 12 hr at 37°C, where the percentage of infected cells were 84.7% and 79.3% as detected by Ad.GFP in Eca109 and TE-1, respectively.

### Quantitative reverse transcription polymerase chain reaction (qRT-PCR)

Total RNA was extracted from 100 mg tissues or 1 × 10^5 ^cells using the RNeasy RNA Mini Kit (Qiagen). First strand cDNA was synthesized using POWERSCRIPT reverse transcriptase (Clontech). The following gene-specific primer pairs were used for quantitative PCR:

*PRAF3*: Forward, 5'- TCATGTTGGCGAGCTATTTCC -3';

Reverse, 5'- GGTTCCGAAGTCTCAACGATG-3'.

*GAPDH*: Forward, 5'- GCTGAGTATGTCGTGGAGTC -3';

Reverse, 5'- AGTTGGTGGTGCAGGATGC -3'.

PCR was performed using a Fast Start Master SYBR Green Kit (Roche) on a LightCycler (Roche). The expression level of target gene mRNA was analyzed using RealQuant software (Roche) and normalized to that of *GAPDH *mRNA.

### Cell lysis and western blot

For preparation of membrane and cytoplasmic protein samples, the tissue or cell samples were harvested in hypotonic lysis buffer (10 mM Tris-HCl, pH 7.5, 10 mM NaCl, 0.2 mM EDTA, 1 mM DTT) supplemented with inhibitors (25 mM b glycerol-phosphate, 25 mM NaF, 1 mM Na_3_VO_4_, 1 mM PMSF, 1 mM benzamidine,). Cell lysates were prepared by Dounce homogenisation and centrifuged at 500 g for 5 min to eliminate nuclei and debris. The supernatant was subjected to ultracentrifugation at 20,000 g for 60 min using the TLA-100.2 fixed angle rotor in Optima TL-100 ultracentrifuge (Beckman). The supernatant (cytoplasm) was adjusted to 100 mM NaCl and 0.5% Nonidet P-40. The membrane pellet was resolubilised in NETN buffer (50 mM Tris-HCl, pH 7.5, 100 mM NaCl, 200 mM EDTA, 0.5% Nonidet P-40, 1 mM DTT, supplemented with inhibitors).

Equal amounts of protein (50 μg) were separated by 10% SDS PAGE and then transferred to nitrocellulose membranes (NY) by electroblotting. The membranes were blocked with 5% BSA in TBST (10 mM Tris-HCl, pH 8.0, 150 mM NaCl, and 0.05% Tween 20) for 1 hr, and then incubated with mouse anti-human specific antibodies overnight at 4°C before subsequent incubation with horseradish peroxidase-conjugated goat anti-mouse antibody (BD Biosciences) for 1 hr at 37°C. Protein was visualized using enhanced chemiluminescence reagent (Santa Cruz). The expression level of target protein was analyzed using LabWork 4.0 program (UVP) and normalized to that of β-actin protein.

### Zymography analysis

The activity of MMP-2 and MMP-9 was detected by zymography analysis. The supernatants of ESCC cells (1.0 × 10^6^) were mixed with sample buffer (10 mM Tris-HCl, pH 7.5, 10 mM NaCl, 0.2 mM EDTA) without reducing agent or heating. The sample was loaded into a gelatin (1 mg/ml) containing SDS-polyacrylamide gel and separated by PAGE. Then, the gel was washed with 2.5% TritonX-100 to remove SDS, rinsed with 50 mM Tris-HCl, pH 7.5, and subsequently incubated overnight at room temperature with the developing buffer (50 mM Tris-HCl, pH 7.5, 5 mM CaCl_2_,1 mM ZnCl_2_, 0.02% thimerosal, 1% Triton X-100). The zymographic activities were revealed by LabWork 4.0 program after staining with 1% Coomassie Blue.

### Apoptosis assay

Cell apoptosis was analyzed by annexin V-FITC assay. Briefly, cells were treated with RNase A (50 mg/ml) for 30 min at 37°C and then stained with annexin V-FITC and propidium iodide using the ANNEXIN V-FITC Kit (Beckman) according to the manufacturer's protocol and subjected to flow cytometric analysis. Viable cells were unstained by annexin V or propidium iodide, early apoptotic cells were stained by annexin V but not propidium iodide, and late apoptotic cells were stained by annexin V and propidium iodide.

### Caspases activity assay

ApoAlert caspase fluorescent and colorimetric assays (BD Biosciences) were used to measure the activity of caspase-8 and caspase-9. Cells (1.0 × 10^6^) were collected and lysed in ice-cold buffer by homogenating. Caspase activity in the supernatant was determined by cleavage of the specific chromophore-conjugated substrates. The substrate peptides of caspase-8 (IETD) and caspase-9 (LEHD) were conjugated to p-nitroaniline (p-NA), and 7-amino-4-methyl coumarin (AMC), respectively. Caspase-8 activity was determined by absorbance of p-NA at 405 nm in a microplate reader. The release of AMC for caspase-9 was measured by quantifying fluorescent intensity in a fluorescence spectrophotometer.

### Wound healing assay

ESCC cells were seeded on 6-well plates at a density of 5 × 10^5 ^cells/well. After the cells reached sub-confluence, the mono-layer cells were wounded by scraping off the cells and then grown in medium for 48 h. The migrated distance of cells was monitored and imaged under a microscope. The distances of cell migration were calculated by subtracting the distance between the lesion edges at 48 hr from the distance measured at 0 h. The relative migrating distance of cells is measured by the distance of cell migration/the distance measured at 0 h.

### Transwell assay

Cell migration and invasion were determined using a transwell (Costar) with a pore size of 0.8 μm. 1 × 10^5 ^cells were seeded in serum-free medium in the upper chamber (normal chamber for migration assay and matrigel-coated chamber for invasion assay), while medium containing 10% FBS in the lower chamber. After incubating for 8 hr at 37°C, cells in the upper chamber were carefully removed with a cotton swab and the cells that had traversed to reverse face of the membrane were fixed in methanol, stained with Giemsa, and counted.

### Statistical Analyses

Statistical significance was tested using SPSS15.0 software. For comparisons of clinical features except for pathological grading between PRAF3 positive and negative groups, chi-square test was performed. The correlation between PRAF3 expression and pathological grade was analyzed by Cochran-Mantel-Haenszel Statistics. Other data are presented as mean ± SEM, using student *t *tests for 2-group comparisons. A *P *value less than 0.05 is considered statistically significant.

## Results

### PRAF3 expression is down-regulated in ESCC specimens and is correlated with pathological grade, tumor stage and lymph node metastasis

**Figure 1:**
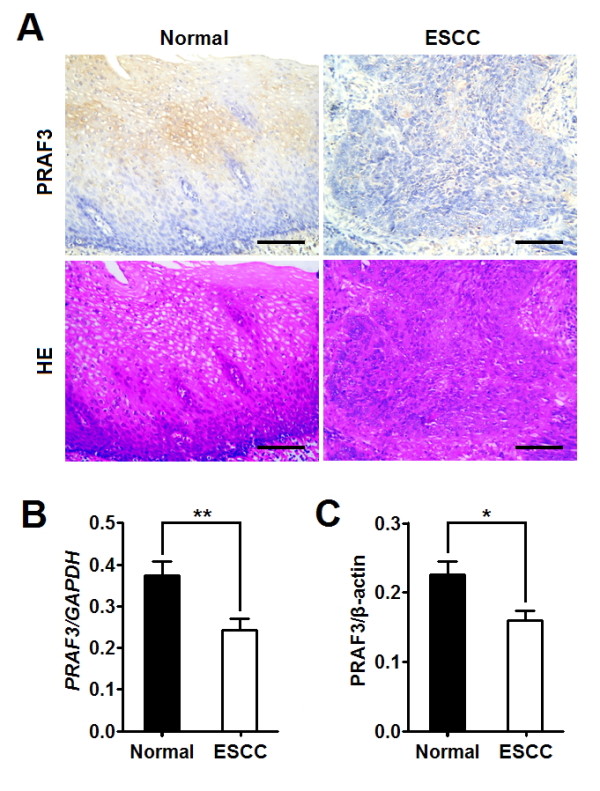
**PRAF3 expression is down-regulated in ESCC specimens as compared to matched normal tissues**. **(A) **Representative images of immunohistochemical staining for PRAF3 in normal esophageal epithelium and esophageal squamous cell carcinoma. Expression level of PRAF3 protein was moderate in normal esophageal epithelium, whereas it was weak in ESCC. Bars = 100 μm. **(B, C) **The expression of PRAF3 mRNA and protein in the ESCC specimens and the matched normal tissues was detected by qRT-PCR and western blot and normalized to that of *GAPDH *and β-actin, respectively. Results showed that the expression level of PRAF3 mRNA **(B) **and protein **(C) **was significantly down-regulated in tumor tissue compared with the matched normal tissue. Data represent mean ± SEM from 57 paired specimens; *, *P *< 0.05, **, *P *< 0.01 by paired t test.

In the present study, we evaluated the expression of PRAF3 in ESCC and the matched normal esophageal tissues by immunohistochemical analysis. It was found that PRAF3 was weakly expressed in ESCC tissues, whereas it was moderately expressed in normal esophageal epithelium (Figure 1A). Through qRT-PCR and western blot, we revealed that the expression level of PRAF3 mRNA and protein was significantly down-regulated in tumor tissue compared with the matched normal tissue (Figure 1B and 1C). These data suggest that down-regulation of PRAF3 might be associated with esophageal tumorigenesis.

We further analyzed the relationship between PRAF3 expression and the clinical features including age, gender, pathological grade, tumor location, tumor stage and lymph node metastasis in ESCC. It was found that the expression of PRAF3 was significantly correlated with pathological grade (*P *= 0.0498), tumor stage (*P *= 0.0208) and lymph node metastasis (*P *= 0.0343); while there was no significant correlation between PRAF3 expression and other factors (Table 1). These results suggest that the PRAF3 might be involved in the progression and metastasis of ESCC.

**Table 1:** Relationship between the expression of PRAF3 and clinical features

clinical features	esophageal squamous cell carcinoma			
	
	PRAF3-positive n (%)	PRAF3-negative n (%)	test	*P*
**Total**	24	33		

**Age (year)**				

< 60	13/24 (54.2)	19/33 (57.6)	0.0656	0.7979

≥ 60	11/24 (45.8)	14/33 (42.4)		

**Gender**				

Male	17/24 (70.8)	25/33 (75.8)	0.1738	0.6768

Female	7/24 (29.2)	8/33 (24.2)		

**Pathological grading**				

Well	11/24 (45.9)	6/33 (18.2)	5.9991	0.0498

Moderately	8/24 (33.3)	12/33 (36.4)		

Poorly	5/24 (20.8)	15/33 (45.4)		

**Tumor location**				

Upper 1/3 middle 1/3	15/24 (62.5)	23/33 (70.0)	0.3239	0.5693

Lower 1/3	9/24(37.5)	10/33 (30.0)		

**Tumor stage**				

T1/T2	13/24 (54.2)	8/33 (24.2)	5.347	0.0208

T3/T4	11/24 (45.8)	25/33 (75.8)		

**Lymph node metastasis**				

Negative	14/24 (58.3)	10/33 (30.3)	4.4785	0.0343

Positive	10/24 (41.7)	23/33 (69.7)		

### Overexpression of PRAF3 induces apoptosis of ESCC cells

**Figure 2:**
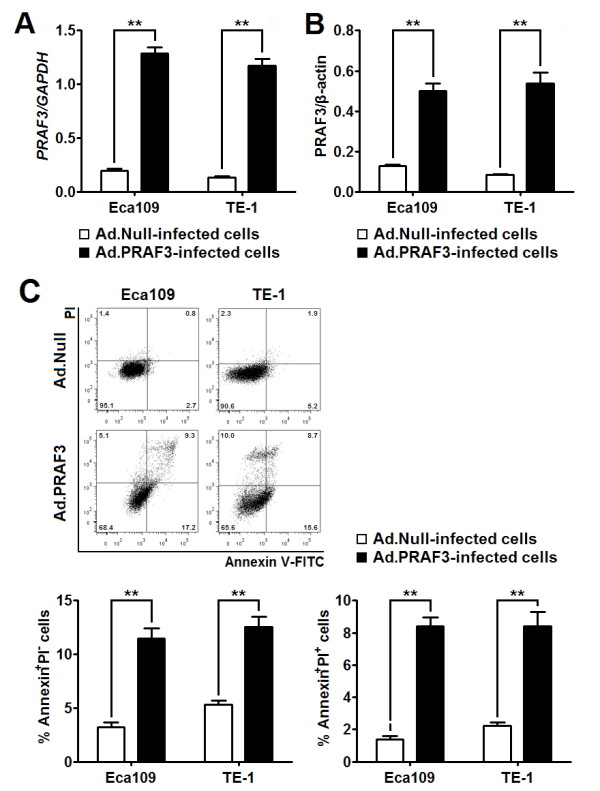
**Overexpression of PRAF3 induces apoptosis of ESCC cells**. Eca109 and TE-1 cells were infected with Ad.PRAF3 (or Ad.Null) at a MOI of 100. The expression of PRAF3 and its effect on cell apoptosis were detected at 72 hr post-infection. **(A, B) **The expression level of PRAF3 mRNA and protein was detected by qRT-PCR and western blot and normalized to that of GAPDH and β-actin, respectively. Histogram showed the expression level of PRAF3 mRNA **(A) **and protein **(B) **in Ad.PRAF3 -infected and Ad.Null -infected ESCC cells. **(C) **Cells were stained with annexin V-FITC and propidium iodide (PI). Flow cytometric contour plots showed the percentage of stained cells. Histogram showed the percentage of Annexin V^+^/PI^- ^and Annexin V^+^/PI^+ ^cells of Ad.PRAF3-infected and Ad.Null-infected ESCC cells. Data represent mean ± SEM from 4 independent experiments; **, *P *< 0.01 by t test.

PRAF3 was reported to be required for the arsenic trioxide and C/EBP-α induced apoptosis [8,9], however the effect of PRAF3 alone on tumor cell apoptosis has not been mentioned before. Here we employed Eca109 and TE-1 (Both derived from well-differentiated ESCCs [14,15]) as model system to evaluate the effect of PRAF3 on the cell apoptosis through adenovirus mediated PRAF3 gene transfer. It was found that the expression of PRAF3 mRNA and protein was significantly increased in the ESCC cells treated with Ad.PRAF3 as compared to the cells treated with Ad.Null at 72 hr post-infection (Figure 2A and 2B). Using annexin V-FITC assay, we found that in cell lines (Eca109 and TE-1), the percentage of annexin V-FITC^+^/PI^- ^and annexin V-FITC^+^/PI^+ ^in Ad.PRAF3-infected cells were both significantly higher than those in Ad.Null-infected cells (Figure 2C). These data indicate that overexpression of PRAF3 promotes apoptosis of ESCC cells.

### PRAF3 induces ESCC cell apoptosis through both caspase-8 and caspase-9 dependent pathways

**Figure 3:**
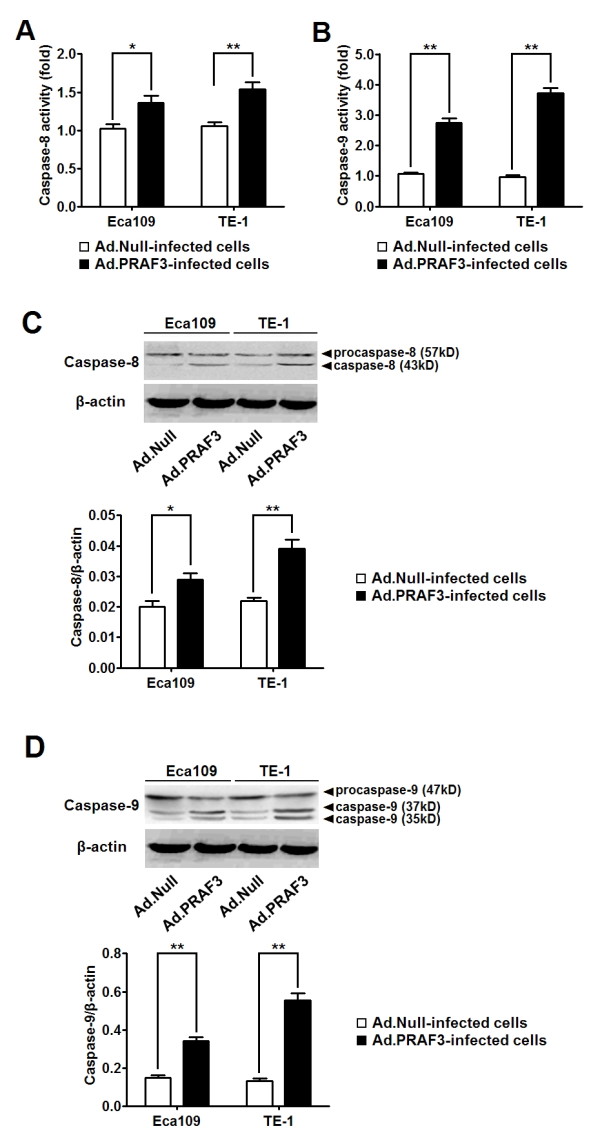
**Overexpression of PRAF3 enhances the activity of caspase-8 and caspase-9 in ESCC cells**. 1.0 × 10^6 ^Eca109 and TE-1 cells were infected with Ad.PRAF3 (or Ad.Null) at a MOI of 100. The activity and expression of caspase-8 and caspase-9 were detected at 72 hr post-infection. **(A, B) **The activity of caspase-8 and caspase-9 were detected by ApoAlert caspase colorimetric and fluorescent assays, respectively. The activity of the Ad.Null-infected cells at 0 hr was defined as 1. Histogram showed the activity of caspase-8 **(A) **and caspase-9 **(B) **in Ad.PRAF3-infected and Ad.Null-infected ESCC cells. **(C, D) **The expression of caspase-8 and caspase-9 were detected by western blot and normalized to that of β-actin. Photographs showed the western blot products and histograms showed the expression of caspase-8 **(C) **and caspase-9 **(D) **in Ad.PRAF3-infected and Ad.Null-infected ESCC cells. Data represent mean ± SEM from 4 independent experiments; *, *P *< 0.05, **, *P *< 0.01 by t test.

Caspase-8 and caspase-9 are identified as the principal upstream caspase effectors in death receptor pathway and death receptor-independent pathway of cell apoptosis, respectively [16,17]. To investigate the mechanism of PRAF3 in the regulation of ESCC cell apoptosis, we evaluated the activity of caspase-8 and caspase-9 in Eca109 and TE-1 cells infected with Ad.PRAF3 (or Ad.Null). As shown in Figure 3A and 3B, the activity of both caspase-8 and caspase-9 was significantly increased in cells treated with Ad.PRAF3 compared with those cells treated with Ad.Null. Furthermore, western blot analysis showed that overexpression of PRAF3 triggered both caspase-8 and caspase-9 cleavage (Figure 3C). These results indicate that PRAF3 could induce ESCC cell apoptosis through both the caspase-8 and caspase-9 dependent pathways.

### Overexpression of PRAF3 inhibits the migration and invasion of ESCC cells

**Figure 4:**
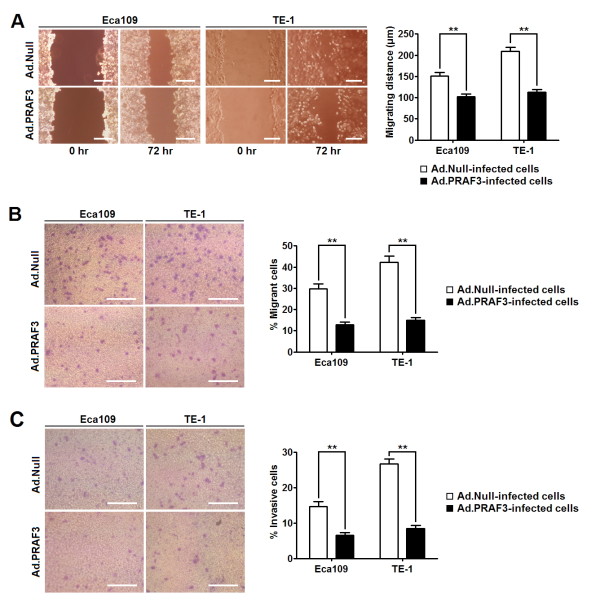
**Overexpression of PRAF3 inhibits the migration and invasion of ESCC cells**. Eca109 and TE-1 cells were infected with Ad.PRAF3 (or Ad.Null) at a MOI of 100. Migration and invasion of cells treated with Ad.PRAF3 (or Ad.Null) were analyzed at 72 hr post-infection. **(A) **Wound healing assay. Photographs represented the cells migrated into the wounded area and histogram showed the relative migration distance of cells. **(B) **Transwell assay. Photographs represented the cells travelled through the micropore membrane and histogram showed the percentage of migrant cells. **(C) **Matrigel-coated transwell assay. Photographs represented the cells invaded through the matrigel and histogram showed the percentage of invasive cells. **Bars = 100 μm**. Data represent mean ± SEM from 4 independent experiments; **, *P *< 0.01 by t test.

To investigate the role of PRAF3 in ESCC metastasis, we detected the migrant and invasive capacity of ESCC cells (Eca109 and TE-1) infected with Ad.PRAF3 or Ad.Null. Through transwell and wound healing assay, we found that the percentage of cells travelled through the micropore membrane was significantly decreased, and the relative migrating distance of cells was significantly shorter in Ad.PRAF3-infected cells as compared to the Ad.Null-infected cells (Figure 4A-D). In addition, by matrigel-coated transwell assay, we revealed that the percentages of Ad.PRAF3-infected Eca109 and TE-1 cells that invaded through the matrigel were significantly lower than those of Ad.Null-infected cells (Figure 4E and 4F). These results indicate that overexpression of PRAF3 inhibits the migration and invasion of ESCC cells.

### PRAF3 suppresses the activity of MMP-2 by integrin a_V_b_3 _signaling in ESCC cells

**Figure 5:**
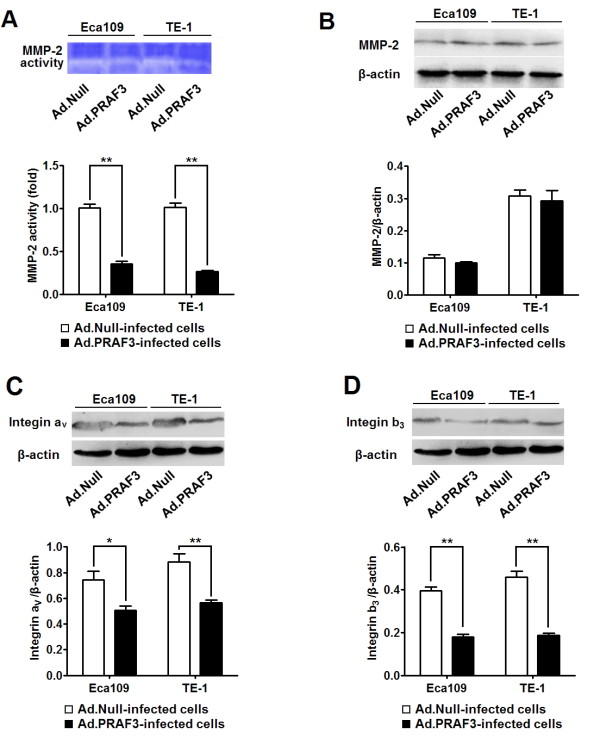
**Overexpression of PRAF3 suppresses the activity of MMP-2 and the expression of integrin aV and b_3_**. 1.0 × 10^6 ^Eca109 and TE-1 cells were infected with Ad.PRAF3 (or Ad.Null) at a MOI of 100. The activity of MMP-2 in supernatants, and the expression of MMP-2, integrin a_V _and integrin b_3 _were detected at 72 hr post-infection. **(A) **The activity of MMP-2 was detected by zymography assay and the activity of the Ad.Null-infected cells was defined as 1. Photographs represented the gelatin zymography and histograms showed the activity of MMP-2 in Ad.PRAF3-infected and Ad.Null-infected ESCC cells. **(B-D) **The expression of MMP-2, integrin aV and integrin b_3 _were detected by western blot and normalized to that of β-actin. Photographs showed the western blot products and histograms showed the expression level of MMP-2 **(B)**, integrin aV **(C) **and integrin b_3 _**(D) **protein in Ad.PRAF3-infected and Ad.Null-infected ESCC cells. Bars = 100 μm. Data represent mean ± SEM from 4 independent experiments; *, *P *< 0.05, **, *P *< 0.01 by t test.

Previous studies reported that MMP-2 was closely related to the metastasis of ESCC [18]. Therefore, we detected the effect of PRAF3 on the activity and expression of MMP-2 in ECA109 and TE-1 ESCC cells. As shown in Figure 5A and 5B, the activity of MMP-2 in the ESCC cells infected with Ad.PRAF3 was significantly decreased in comparison with those cells infected with Ad.Null. However, the expression level of MMP-2 protein was comparable between the two groups (Figure 5B). These results indicate that PRAF3 suppresses the activity but not the expression of MMP-2.

Since it was reported that PRAF3 inhibited melanoma metastasis by integrin a_V_b_3 _signaling [10] and integrin aVb3 could promote tumor metastasis by activating MMP-2 [19], we further analyzed the expression of integrin a_V _and b_3 _subunits in ESCC cells infected with Ad.PRAF3 (or Ad.Null) by western blot. It was found that the expression level of integrin a_V _and b_3 _in Ad.PRAF3-infected cells was significantly lower than that in Ad.Null-infected cells (Figure 5C and 5D). These data suggest that PRAF3 might suppress the activity of MMP-2 by down-regulating integrin a_V_b_3 _signaling in ESCC cells.

### PRAF3 suppresses the expression of MMP-9 by affecting the membranous location of CCR5 in ESCC cells

**Figure 6:**
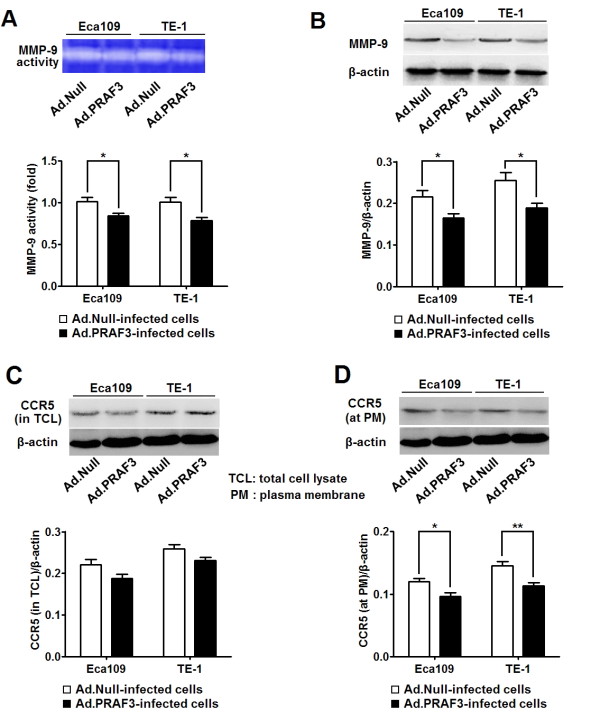
**Overexpression of PRAF3 suppressed the expression and activity of MMP-9 and affects the membranous location of CCR5**. 1.0 × 10^6 ^Eca109 and TE-1 cells were infected with Ad.PRAF3 (or Ad.Null) at a MOI of 100. The activity of MMP-9 in supernatants, and the expression of MMP-9 and CCR5 were detected at 72 hr post-infection. **(A) **The activity of MMP-9 was detected by zymography assay and the activity of the Ad.Null-infected cells was defined as 1. Photographs represented the gelatin zymography and histograms showed the activity of MMP-9 in Ad.PRAF3-infected and Ad.Null-infected ESCC cells. **(B) **The expression of MMP-9 was detected by western blot and normalized to that of β-actin. Photographs showed the western blot products and histograms showed the expression level of MMP-9 protein in Ad.PRAF3-infected and Ad.Null-infected ESCC cells. **(C, D) **The expression of CCR5 in total cell lysate and at plasma membrane were detected by western blot and normalized to that of β-actin. Photographs showed the western blot products and histograms showed the expression of CCR5 in total cell lysate **(C) **and at plasma membrane **(D) **in Ad.PRAF3-infected and Ad.Null-infected ESCC cells. Data represent mean ± SEM from 4 independent experiments; *, *P *< 0.05, **, *P *< 0.01 by t test.

MMP-9 was another matrix metalloproteinase which was closely related to the metastasis of ESCC [18]. Here, we detected the effect of PRAF3 on the activity and expression of MMP-9 in ESCC cells. It was found that both the activity and expression of MMP-9 in Ad.PRAF3-infected cells was significantly lower than those in Ad.Null-infected cells (Figure 6A and 6B). These results suggest that PRAF3 could suppress the activity of MMP-9 by inhibiting its expression.

In light of previous findings that the expression of MMP-9 was up-regulated by CCL5/CCR5 axis [20] and PRAF3 protein contains a CCR5 binding motif [21], we hypothesized that PRAF3 might inhibit the expression of MMP-9 through its regulation of CCR5. To test the hypothesis, we further studied the effect of PRAF3 on the expression of CCR5 in ESCC cells. In the present study, we found that the expression of CCR5 at the plasma membrane was significantly lower in Ad.PRAF3-infected cells as compared to Ad.Null-infected cells (Figure 6C), while the expression of CCR5 in the total cell lysate was comparable between the 2 groups (Figure 6D). These data suggest that PRAF3 could suppress the expression of MMP-9 by affecting the membranous location of CCR5 in ESCC cells.

## Discussion

Members of the Prenylated Rab acceptor domain family (PRAF) are essential for the regulation of many cellular processes [22,23]. Here we have demonstrated that PRAF3 could induce apoptosis and inhibit migration and invasion of ESCC cells and hence may serve as a tumor suppressor in ESCC.

Although the role of PRAF3 has been studied in several other tumors using tumor cell lines [7,9,10,24], there is a lack of investigation into the relationship between PRAF3 expression and the clinical features of ESCCs. In the present study, we found that the expression level of PRAF3 mRNA and protein in ESCC tissues was significantly lower than that in the matched normal tissues. In addition, we found that down-regulation of PRAF3 expression was significantly correlated with poorly differentiated grading, advanced tumor stage and lymph node metastasis of ESCC. These results would imply that PRAF3 may play an important role in regulating the progression and metastasis of ESCCs, although further in vivo studies are needed to substantiate this conclusion. We noticed that the expression of PRAF3 in normal squamous tissue is not homogeneous and appeared as a decreasing gradient from the differentiating squamous compared to the transit amplifying and stem cell compartment. However, there is a lack of information regarding the role of PRAF3 in the regulation of differentiation of normal squamous cells. In this sense, it would be interesting to see whether down-regulation of PRAF3 expression would result in retarded differentiation of normal squamous cells.

Apoptosis is a form of cellular suicide that closely related to the progression and metastasis of tumor cell [25]. Previous studies showed that PRAF3 serves as an important regulator in cell apoptosis [8,9,24]. For example, Zhou et al. demonstrated that the expression of PRAF3 is up-regulated and required for the arsenic trioxide induced tumor cell apoptosis in Hela and MCF-7 [9]. Wang et al showed that PRAF3 is a key mediator in C/EBP-α induced cell apoptosis of HEK293 and NIH3T3 [8]. However, whether changes in PRAF3 expression alone affect tumor cell apoptosis has not been reported. In the present study, we have examined the effect of PRAF3 on the cell apoptosis through adenovirus mediated PRAF3 gene transfer. We demonstrate that overexpression of PRAF3 induces cell apoptosis in human ESCC.

Cell apoptosis mainly involves two signaling pathways: the death receptor pathway and the death receptor-independent (or mitochondrial) pathway [16]. Caspase-8 and caspase-9 have been identified as the key signal molecules of the two pathways respectively [26,27]. In the present study, we showed that overexpression of PRAF3 could significantly increase the activity of both caspase-8 and caspase-9 in ESCC cells, suggesting that PRAF3 could induce ESCC cell apoptosis through both caspase-8 and caspase-9 dependent pathways. Notably, we found that the overexpression of PRAF3 induced greater changes in caspase-9 than caspase-8 expression, suggesting that PRAF3 may induce cell apoptosis mainly through the death receptor-independent pathway in human ESCC.

Tumor metastasis is the leading cause of low survival rate of ESCC patients. Previous studies showed that PRAF3 acts as a down-regulatory factor for cell migration and invasion in melanoma, osteosarcoma, cervical cancer and breast cancer [7,10]. In the present study, we investigated the role of PRAF3 in ESCC metastasis by clinical investigation and cellular experiment. As mentioned above, our clinical investigation suggests that the expression of PRAF3 in ESCC is negatively related to the tumor metastasis. Moreover, with ESCC cell lines infected with Ad.PRAF3, we further demonstrate that overexpression of PRAF3 significantly inhibits the migration and invasion of ESCC cells. Our results indicate that down regulation of PRAF3 may contribute to the metastasis of ESCC.

It is well known that matrix metalloproteinases (MMPs), a family of zinc- dependent endopeptidases, are involved in tumor metastasis in many aspects such as tumor-induced angiogenesis, tumor invasion, and establishment of metastatic foci at the secondary site, etc [28-30]. Among the many MMPs, MMP-2 and MMP-9 are reported to be closely related to the tumor metastasis in ESCC [18]. However, whether PRAF3 regulates the activity of MMP-2 and MMP-9 in ESCC has not been documented. In the present study, we found that overexpression of PRAF3 significantly suppressed the activity of both MMP-2 and MMP-9, suggesting that PRAF3 could inhibit ESCC metastasis partially through the repression of MMP-2 and MMP-9 dependent pathway.

Previous study showed that PRAF3 could suppress the activity of MMP-2 by modulating the integrin a_v_b_3 _signaling in melanoma [10]. Here we show that overexpression of PRAF3 did not alter the expression of MMP-2 but significantly repressed the expression of integrin a_v _and b_3_, suggesting that PRAF3 may inhibit the activity of MMP-2 probably by the inhibition of integrin a_v_b_3 _signaling in ESCC cells.

On the other hand, we found that the expression of MMP-9 was significantly lower in Ad.PRAF3 treated cells than the controls. Since it was reported that the expression of MMP-9 was regulated by CCL5/CCR5 axis [20] and PRAF3 contains a CCR5 binding motif [21], we further studied the affection of PRAF3 on the CCR5. Our data show that overexpression of PRAF3 did not alter the level of CCR5 in the total cell lysate but significantly decreased the level of CCR5 at the plasma membrane. Considering that PRAF3 is a transmembrane protein located at the endoplasmic reticulum [21], we propose that PRAF3 might suppress the traffic of CCR5 from endoplasmic reticulum to plasma membrane, and thereby inhibits the expression and in turn the activity of MMP-9.

## Conclusions

We found that PRAF3 expression in ESCC tissues was significantly lower than that in matched normal tissues and was correlated with the clinical features of pathological grade, tumor stage and lymph node metastasis. Moreover, overexpression of PRAF3 induces apoptosis and inhibits migration and invasion in ESCC cells. Based on these findings, we propose that PRAF3 might be a lead molecule for the development of novel treatment of ESCC.

## Competing interests

The authors declare that they have no competing interests.

## Authors' contributions

SDH and YFT conceived the design of the study and were in charge of its coordination. YY participated in data analysis, performed data interpretation and drafted the manuscript. GZS carried out the apoptosis analysis and helped to draft the manuscript. GJJ carried out the migrant and invasion analysis and helped to draft the manuscript. ZJG and JZ performed Immunohistochemistry and pathologic analysis. DJG performed molecular biology experiment. JT participated in cell culture. All authors read and approved the final manuscript.

## Pre-publication history

The pre-publication history for this paper can be accessed here:

http://www.biomedcentral.com/1471-2407/12/97/prepub
